# Mechanisms of fungal community assembly in wild stoneflies moderated by host characteristics and local environment

**DOI:** 10.1038/s41522-022-00298-9

**Published:** 2022-04-27

**Authors:** Yu-Xi Zhu, Qing-Bo Huo, Tao Wen, Xin-Yu Wang, Meng-Yuan Zhao, Yu-Zhou Du

**Affiliations:** 1 School of Horticulture and Plant Protection & Institute of Applied Entomology, Yangzhou University, Yangzhou, 225009 China; 2 Joint International Research Laboratory of Agriculture and Agri-Product Safety, the Ministry of Education, Yangzhou University, Yangzhou, 225009 China; 3 The Key Laboratory of Plant Immunity, Jiangsu Provincial Key Lab for Organic Solid Waste Utilization, Jiangsu Collaborative Innovation Center for Solid Organic Wastes, Educational Ministry Engineering Center of Resource-saving fertilizers, Nanjing Agricultural University, Nanjing, 210095 China

**Keywords:** Microbiome, Microbial ecology

## Abstract

Deterministic and stochastic forces both drive microbiota assembly in animals, yet their relative contribution remains elusive, especially in wild aquatic-insect-associated fungal communities. Here, we applied amplicon sequencing to survey the assembly mechanisms of the fungal community in 155 wild stonefly individuals involving 44 species of 20 genera within eight families collected from multiple locations in China. Analysis showed that fungal diversity and network complexity differed significantly among the eight stonefly families, and that the fungal communities in stoneflies exhibited a significant distance-decay pattern across large spatial scales. Both a structural equation model and variance partitioning analysis revealed that environmental factors (e.g., geographical, climatic) outweigh host attributes in shaping the fungal community of stoneflies. Using neutral and null model analyses, we also find that deterministic processes play a larger role than stochasticity in driving the fungal community assembly. However, the relative contribution of ecological processes including dispersal, drift, and selection, varied strongly with host taxonomy. Furthermore, environmental conditions also significantly affect the strength of these ecological processes. Overall, our findings illustrate that variations in host attributes and environment factors may moderate the relative influence of deterministic and stochastic processes to fungal community composition in wild stoneflies, which provides new insights into mechanisms of microbial community assembly in aquatic arthropods.

## Introduction

Stoneflies (Plecoptera) are one of the smallest and most ancient orders of insects, with approximately 3,700 species in 16 families worldwide^1^. Nymphs of the vast majority of species live mainly in streams and feed on leaves or other invertebrates, while adults are terrestrial and generally shredders of leaves^2^. Stoneflies are widely renowned as a key indicator of water quality and a flagship environmental parameter^3^. Despite this importance, several species remain threatened with extinction due to water pollution, climate change, habitat alterations, and other environmental factors^4^. To protect these vital species requires and in-depth understanding of their biology, but also understanding of species that are tightly associated with them. In particular, host-associated microbiota perform vital functions for the stonefly^5^, and changes to their homeostasis might be disrupted during rapid shifts in the host habitat environment and indirectly affect host health^6^. Deciphering the mechanisms underlying microbiota community assemblage is essential to stonefly conservation, especially over evolutionary and ecological timescales.

Community assemblies are determined by four basic processes: diversification, selection, drift, and dispersal^7^. Multiple deterministic factors (e.g., selection) have been shown to modulate microbial community diversity patterns in many animals^8–11^. Specifically, the host is a major force in governing microbial community structures in several animal species^12–16^. In parallel, environmental factors such as diet^17–19^, climate, and geographic variables^20,21^ are also important. The relative contribution of these two types of factors in shaping the host-microbiota remains highly controversial^22–24^. In contrast, neutral community assembly theory emphasizes the importance of stochastic processes (e.g., drift, dispersal limitation) in governing microbial communities^25,26^. While stochastic assembly is often neglected in studies in microbial ecology^27,28^, it has been revealed to work simultaneously with deterministic processes in driving microbial communities in a broad range of natural systems. The relative contribution of each ecological process varies among different host taxa and different environments^19,29–31^. For instance, strong effects of deterministic forces on gut microbial community composition have been reported in studies on wild *Drosophila*^32^, while neutrality is dominant over selection in the differentiation of the gut bacterial community in honeybees^33^. As these works demonstrate, the microbial community assembly in terrestrial insects has received some attention^32–35^. However, to our knowledge, no studies have systematically evaluated the relative importance of deterministic and stochastic processes in aquatic insect mycological assemblies across large geographical areas. Stoneflies, which are not only an ecologically critical taxa but also exhibit ecological specialization over a broad habitat range, offer an excellent system for investigating microbial community assembly within an environmental and geographic context.

Here, we report on a comprehensive survey of fungal microbiomes in natural populations of stoneflies using a fungal ITS genes high-throughput sequencing. The work involves 155 individuals from 52 populations sampled from multiple locations across China and represents 44 species and 20 genera from eight families within Plecoptera. The purpose of the study was to (i) investigate whether variation in fungal composition is related to host condition and multiple environmental factors, and (ii) clarify the mechanisms underlying fungal community assembly in wild stoneflies. We first examined the patterns of stonefly fungal communities across different subgroups and large geographic regions. We then conducted neutral and null model analyses to quantitatively decipher the relative contribution of ecological processes including selection, dispersal, and drift in driving the assembly of stonefly-associated fungal communities. Lastly, we explored the differences of these processes across spatial scales.

## Results

### Fungal community diversity and structure among stonefly taxa

**Fig. 1:**
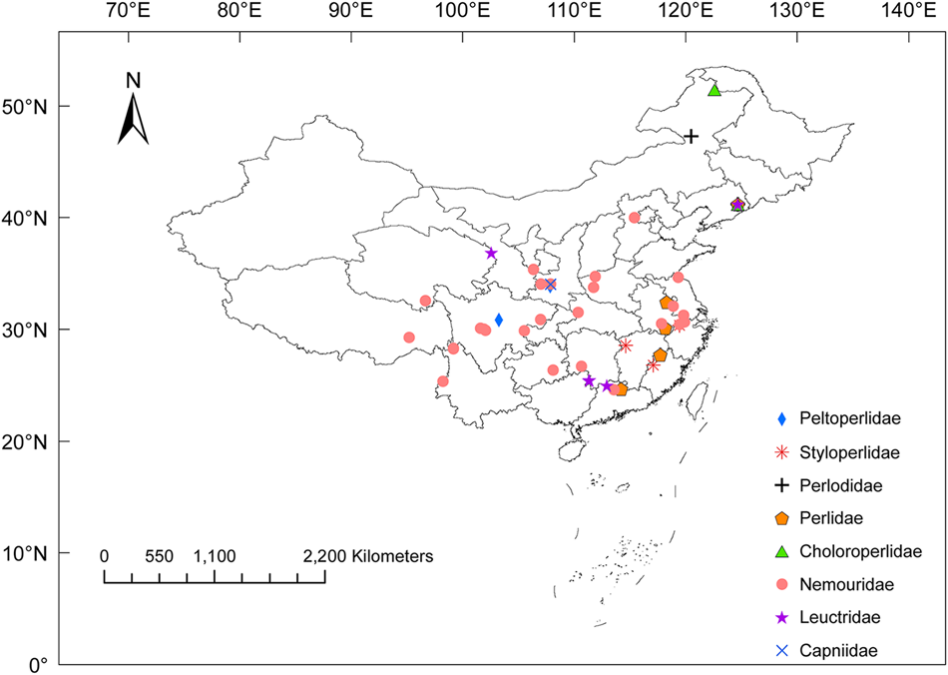
Sampling locations of wild stoneflies across multiple geographic regions of China. Differently colored symbols represent different stonefly families. The figure was generated using ArcGIS 10 Crack software based on a template map from the Chinese National Basic Geographic Information Center (http://ngcc.sbsm.gov.cn).

**Fig. 2:**
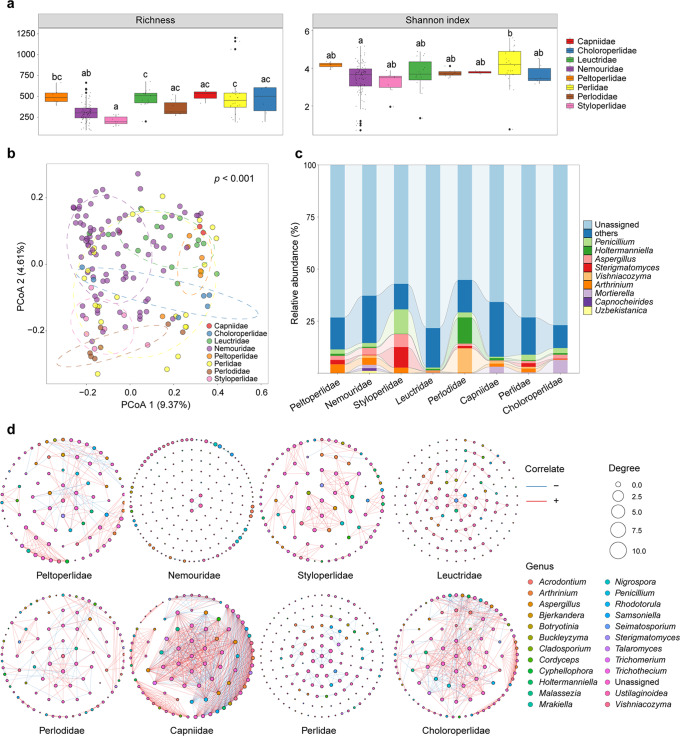
Fungal community diversity and composition among eight stonefly families. **a** Richness and Shannon indices of different stonefly fungal communities. Different letters denote significant differences between stonefly families with ANOVA tests (*p* < 0.05). **b** Principal coordinate analysis (PCoA) of fungal community Bray-Curtis dissimilarities with permutational analysis of variance among different stonefly families. The variation explained by each principal coordinate is denoted in parentheses. **c** Relative abundances of each fungal genus in different stonefly families. **d** Co-occurrence networks of the fungal communities from each stonefly family based on Spearman’s correlation analysis between OTUs. Blue and red lines represent significant negative and positive correlations, respectively. The sizes of the points indicate the relative abundance of OTUs in each microbial community.

A total of 6,675,681 clear reads with an average of ~ 43,068 reads per sample were obtained from 155 individuals from 20 genera belonging to eight families after filtering for quality and removal of samples with low read numbers (Fig. 1; Supplementary Table 1). The α diversity of the fungal community significantly differed among eight stonefly families (Kruskal-Wallis test: Richness, *p* < 0.05; Shannon index, *p* < 0.05), with the highest Shannon index observed in Perlidae (Fig. 2a). Principal coordinate analyses (PCoA) based on the Bray-Curtis distance matrix revealed that compositions of the fungal communities varied significantly among stonefly taxa (perMANOVA: *F* = 3.55, *R*^*2*^ = 0.14, *p* < 0.001; Fig. 2b). Taxonomic assignment revealed that the majority of OTUs (~80%) were unassigned. Of the assigned taxa, the relative abundance of the dominant fungal genera, such as *Aspergillus*, *Holtermanniella*, *Penicillium*, and *Sterigmatomyces*, differed among eight stonefly families (Fig. 2c). The most prevalent fungal genus in all sampled stoneflies was *Aspergillus* (44.52%), which we identify as a core member of mycological communities in wild stoneflies.

Network analyses showed that co-occurrence of species exhibited different patterns among eight stonefly families, with more connections in fungal communities from Capniidae (872 edges, 592 positive and 280 negative) and Choloroperlidae (555 edges, 377 positive and 178 negative) compared to the other six families (Fig. 2d). Of the set of network topological features calculated, network complexity, indicated by the average degree value, was noticeably higher in Capniidae (19.16) and Choloroperlidae (11.1) than in Leuctridae (2.23), Perlidae (2.32) and Nemouridae (1.45; Table 1). In addition, the taxonomic composition of the networks differed among eight stonefly families, and in most stonefly families some hub nodes were unassigned (Fig. 2d).

**Table 1:** Topological properties of co-occurrence networks for each stonefly family.

Stonefly family	Edges	Positive edges	Negative edges	Vertices	Connectance	Average degree	Average path length	Diameter	Clustering coefficient	Clusters	Centralization degree	Centralization betweenness	Centralization Closeness
Peltoperlidae	244	169	75	84	0.070	5.810	4.318	9.509	0.727	5	0.075	0.111	0.018
Nemouridae	30	30	0	41	0.037	1.463	1.171	2.457	0.750	17	0.038	0.004	0.003
Styloperlidae	179	167	12	90	0.045	3.978	2.337	6.010	0.892	15	0.045	0.025	0.004
Leuctridae	77	76	1	71	0.031	2.169	2.043	5.175	0.664	19	0.069	0.009	0.003
Perlodidae	215	143	72	92	0.051	4.674	3.752	10.084	0.490	8	0.124	0.126	0.015
Capniidae	832	548	284	91	0.203	18.286	1.000	1.000	1.000	6	0.097	0	0.003
Perlidae	80	80	0	72	0.031	2.222	1.255	2.534	0.844	23	0.053	0.002	0.001
Choloroperlidae	549	356	193	101	0.109	10.871	2.731	7.628	0.629	7	0.161	0.060	0.014

### The geographic distance-decay pattern of fungal community composition

**Fig. 3:**
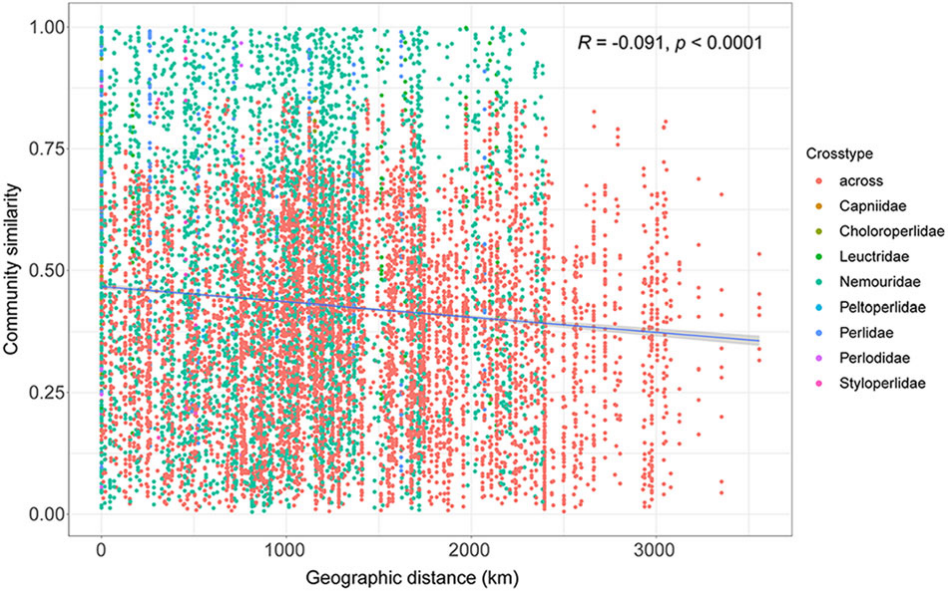
Geographic patterns in stonefly fungal communities. Distance-decay curves of fungal communities based on Bray–Curtis similarities in wild populations of stoneflies. The line represents the ordinary least-squares linear regression.

We further investigated spatial variation in stonefly fungal communities. In communities that span across multiple geographic regions, significant distance-decay relationships between fungal Bray–Curtis similarities and geographical distances were detected overall (*R* = −0.091, *p* < 0.0001) (Fig. 3).

### The relation between host-related and environmental variables and fungal community composition

**Fig. 4:**
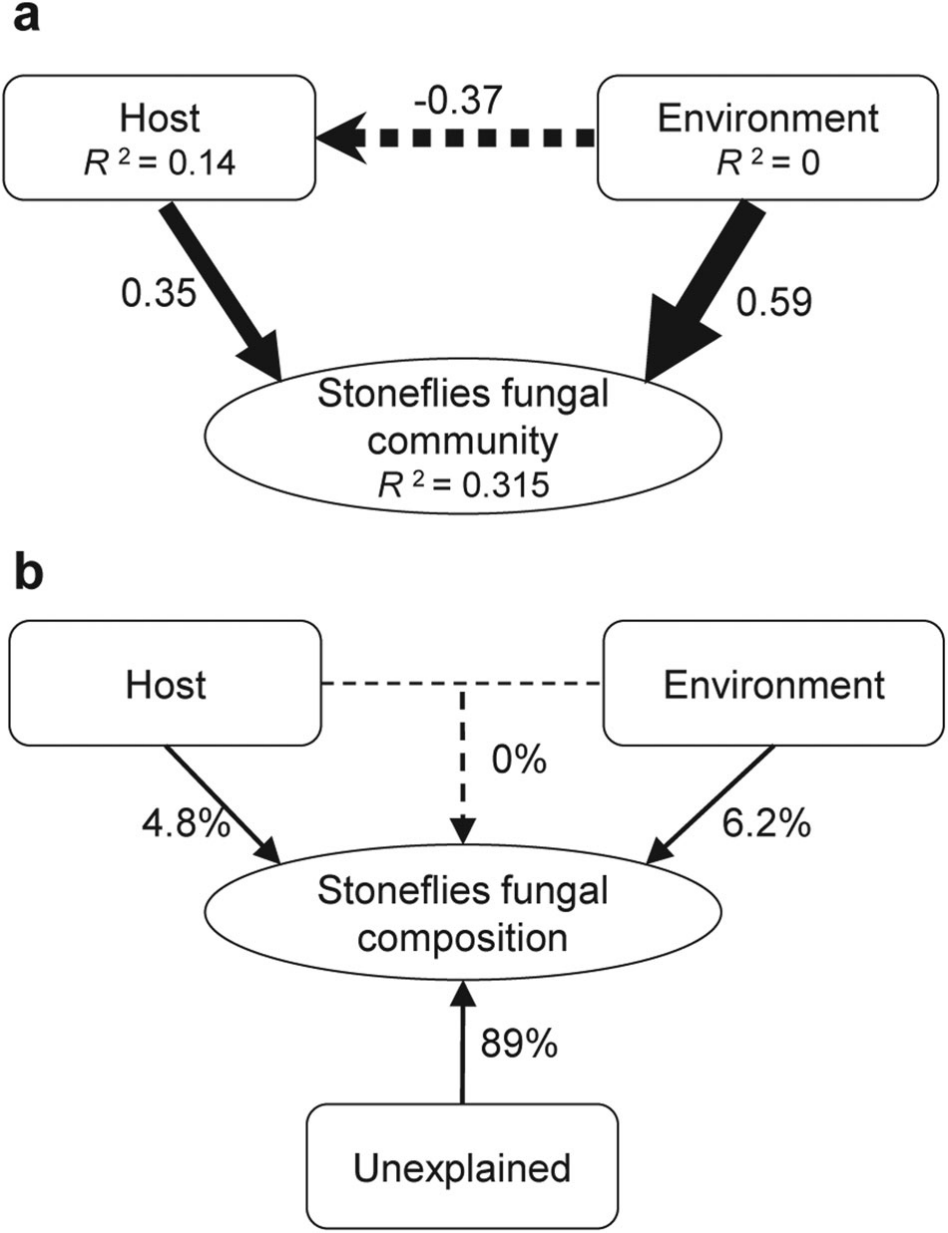
The effect of host-related and environmental factors on the stonefly fungal community composition. **a** Structural equation model provided insight on connections between both host-related and environmental variables to fungal community composition. The blue and red arrows indicate statistically significant negative and positive paths, respectively. The width of the arrows represents the strengths of these relationships. The *R*^*2*^ values under each box indicate the amount of variation in that variable explained by the input arrows. Numbers next to arrows are unstandardized slopes. **b** Variance partitioning analysis (VPA).

A structural equation model (SEM) was used to explore the correlation between fungal community variation and both host-related and environmental variables. Results showed that fungal community structure was directly and significantly impacted by host-related and environmental variables, and their path coefficients were 0.35 and 0.59, respectively (Fig. 4a). Moreover, variance partition analysis (VPA) showed that host-related factors explained only 4.8% of fungal community composition and environmental variables only 6.2% (Fig. 4b). This leaves the large majority of the compositional variance (89%) unexplained (Fig. 4b), suggesting that complex processes govern fungal community assembly.

We then estimated the relative contribution of multiple environmental factors including location, altitude, latitude, longitude, annual mean temperature (AMT), and annual mean precipitation (AP), to overall fungal community compositional variation. Among these variables, the fungal community was the most significantly impacted by AP (explained variation: 1.47%; *F* = 1.61, *p* < 0.001), followed by AMP (1.30%; *F* = 1.70, *p* < 0.001), altitude (1.28%; *F* = 1.76, *p* < 0.001), latitude (1.14%; *F* = 1.57, *p* < 0.001), longitude (1.10%; *F* = 1.60, *p* < 0.001), and location (0.88%; *F* = 1.35, *p* < 0.001) (Supplementary Fig. 1; Supplementary Table 2).

### Ecological processes governing stonefly fungal community assembly

**Fig. 5:**
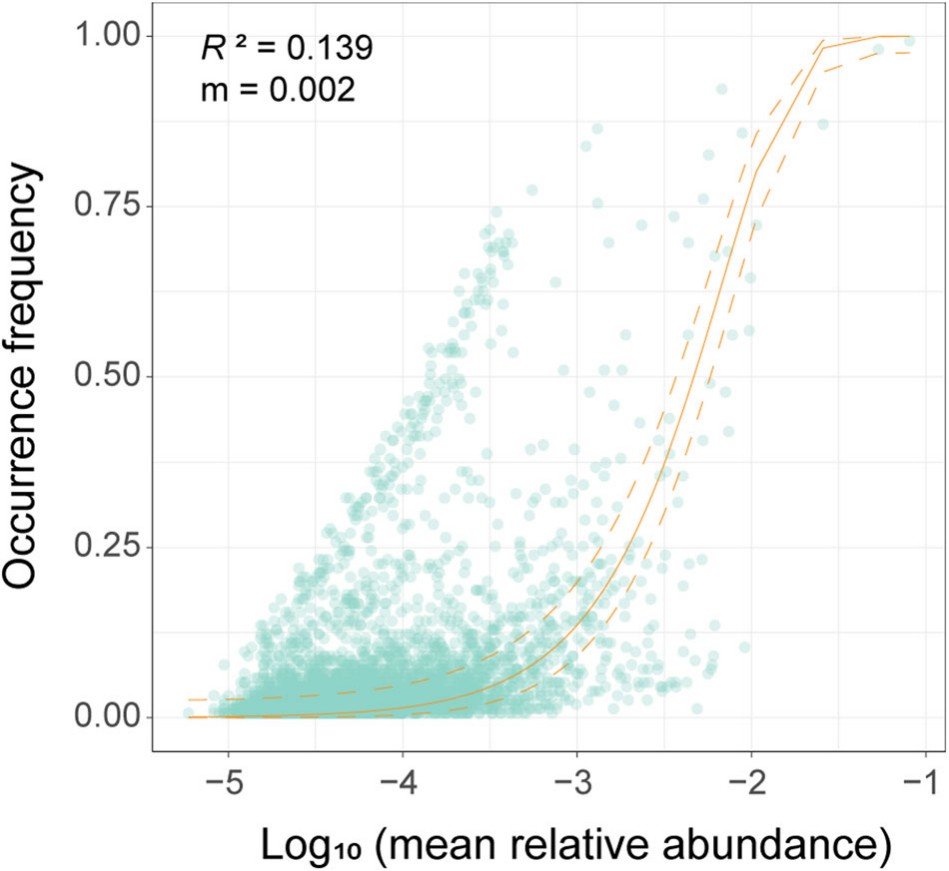
Fit of a neutral model on fungal community assembly in stoneflies. The yellow solid and dashed lines indicate the predicted occurrence and 95% confidence interval of the neutral model, respectively. *R*^2^ indicates the goodness of fit to the neutral model, and *m* shows the migration rate.

To explore the role of neutral processes in determining stonefly fungal communities, we first deployed the Sloan neutral model to assess all samples and also each stonefly family separately. For all stonefly samples, the frequency of fungal OTUs within metacommunities of stoneflies fit rather weakly to the neutral model, and the majority of OTUs fell outside of the 95% confidence interval of the neutral model prediction (*R*^*2*^ = 0.139, *m* = 0.002) (Fig. 5). This indicates that deterministic processes play a more critical role than stochasticity in the formation of stonefly fungal communities. However, the degree of influence that neutral processes have on fungal community assembly differed among stonefly families (Supplementary Fig. 2).

**Fig. 6:**
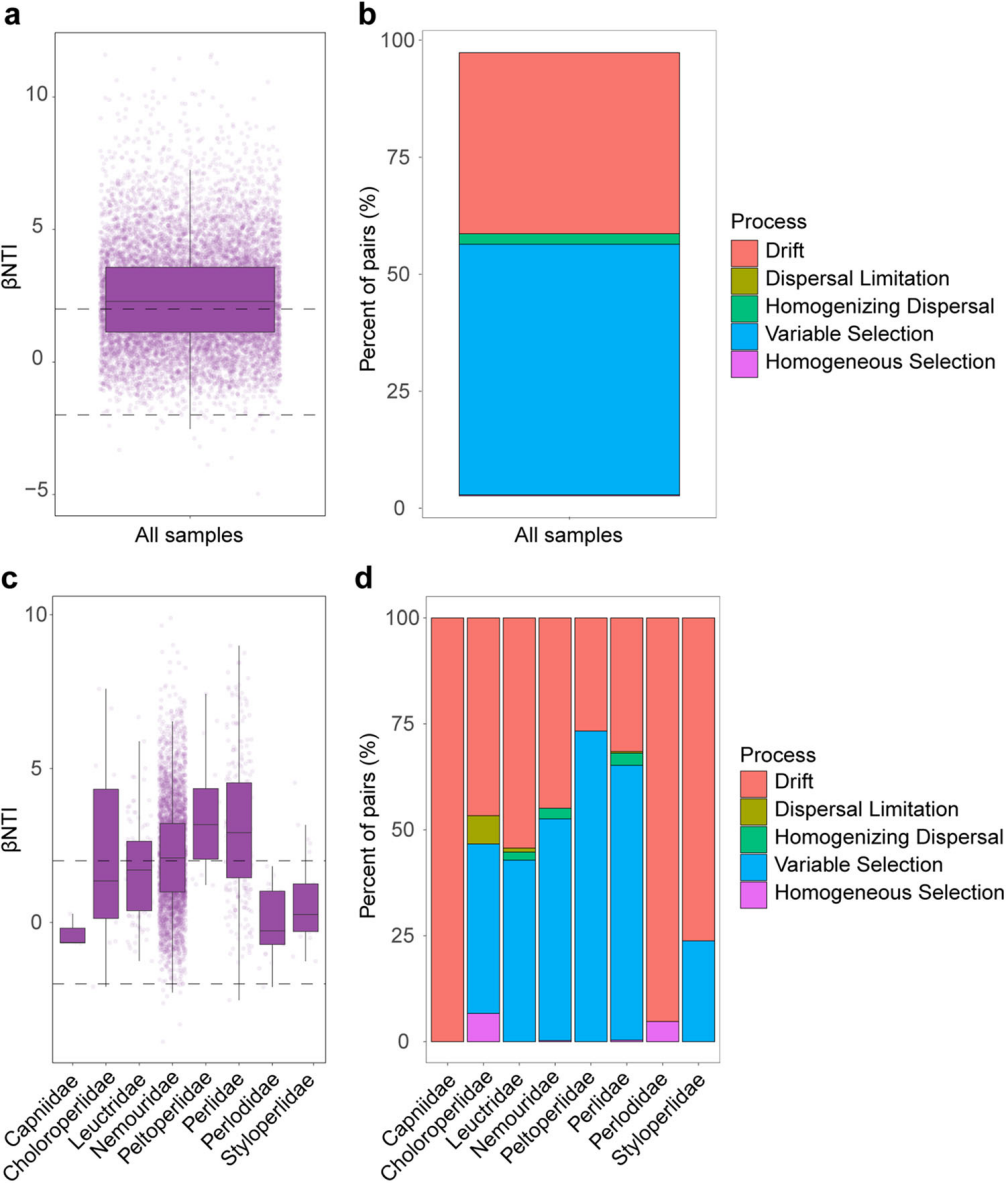
Mechanisms of stonefly fungal community assembly evaluated using null model analysis. Contributions of deterministic (|βNTI | ≥ 2) and stochastic processes (|βNTI | < 2) on fungal community assembly in all stoneflies (**a**) and in each of eight stonefly families (**c**). The relative contribution of ecological processes (i.e., homogeneous selection, heterogeneous selection, homogenizing dispersal, dispersal limitation, and drift) in driving the fungal assembly in all samples (**b**), and in each stonefly family (**d**).

As a second approach, the null model was used to quantify the relative impact of stochastic and deterministic forces in shaping fungal community assembly. When disregarding taxonomic information, we found that variable selection, a deterministic process, was the top factor in influencing fungal community assembly (βNTI > 2, relative contribution 56.58%; Fig. 6a), followed by drift (40.86%) and homogenizing dispersal (2.35%) (Fig. 6b). Upon examining individual stonefly families, influence of each ecological process varied among groups (Fig. 6c): drift was the primary process governing the fungal communities from five families (Capniidae, Perlodidae, Styloperlidae, Leuctridae, and Choloroperlidae) to divergence, while the process of variable selection dominated in driving the fungal communities from Nemouridae, Petloperlidae and Perlidae to convergence. In contrast, dispersal limitation and homogenizing dispersal had a negligible degree of influence on fungal community assembly in the majority of stoneflies (Fig. 6d). These results suggest that a combination of variable selection and drift, their relative influence strongly dependent on host taxa, drive the assembly of stonefly fungal communities.

### Environmental variables affect fungal community assembly

**Fig. 7:**
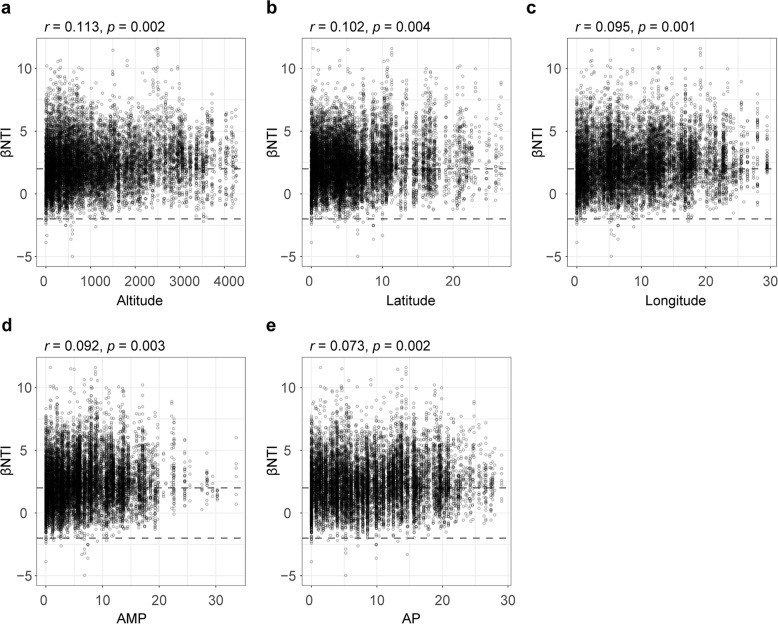
Effects of environmental factors on stonefly fungal community assembly. Mantel analysis used to evaluate the correlation between the β-Nearest Taxon Index (βNTI) and the multiples environmental variables: (**a**) altitude, (**b**) latitude, (**c**) longitude, (**d**) annual mean temperature (AMT) and (**e)** annual mean precipitation (AP).

To evaluate the influence of environmental factors on stonefly fungal community assembly, we explored the relation between βNTI value and five environmental factors with the Mantel test. The results revealed that the βNTI value was significantly positively correlated with altitude (*r* = 0.113, *p* = 0.002), latitude (*r* = 0.102, *p* = 0.004), longitude (*r* = 0.095, *p* = 0.001), AMT (*r* = 0.092, *p* = 0.003), and AP (*r* = 0.073, *p* = 0.002) (Fig. 7), indicating that these environmental variables have significant impact on the fungal community assembly in stoneflies.

## Discussion

Disentangling the mechanisms of community assembly is a critical topic in microbial ecology^23,36^. To our knowledge, this work represents the large-scale study investigating fungal community diversity patterns and assembly mechanisms that incorporates the majority of wild stonefly species from various geographic locations in China. We demonstrated that both host-related and environmental factors significantly affect the stonefly fungal community with different relative contribution. In addition, we found that deterministic processes (i.e., variable selection) dominated over stochastic processes (e.g., stochastic drift) in shaping stonefly microbiota composition. The relative contribution of ecological processes varied among stonefly taxa and was also strongly linked to the environment.

### The keystone taxa for stonefly fungal microbiome

In addition to being a valuable method in studying the patterns of host-associated microbial communities, network analysis is also used to detect keystone taxa that have an essential ecological role in microbiome assemblies or crucial ecosystem functions^37,38^. While many keystone taxa identified in our analyses were unassigned (Fig. 1d), the assigned groups revealed some network hub taxa in the stoneflies fungal community belonged to Ascomycota, and of these, *Aspergillus* were particularly abundant in some stonefly families. Similarly, members of Ascomycota have ubiquitous occurrences and have been observed to be a key member of fungal communities in other insects, such as the silkworm^39^ and rice planthoppers^40^, playing a key role in the degradation of plant materials^41^. Due to many stonefly species feeding on leaves^42,43^, we speculate that the higher abundance of *Aspergillus* in stoneflies might be linked with involvement in the degradation of plant cell wall polysaccharides and in turn providing nutrition for hosts. Further research might elucidate the biological or ecological functions of these keystone taxa in wild stoneflies. In addition, we found that fungal network patterns differed among stonefly families. Considering variations in morphology, physiology, and ecology that exist among different stonefly taxa^42–44^, the co-occurrence network patterns of stonefly fungal communities might, at least partially, be dependent on the complex interplay between environmental factors and host history.

### Effects of environmental factors and host attributes on the fungal community of stoneflies

The host plays a primary role in shaping associated microbiota in diverse arthropods^45^. Microbiota composition is known to vary among host species, sex, and developmental stages, and these differences have been generally attributed to host-specific selection^15,32,46^. Host morphology and physiochemical conditions vary among species and developmental stages, impacting the community structure of microbiota^12,16,32^. Thus, despite sympatric or adjacent wild stonefly species sharing the same or similar environmental conditions, various morphological and physiochemical differences may explain their different fungal community composition. Aside from host-related influences, environmental factors, mainly geographic and climatic factors, also affect the fungal composition in stoneflies^20,47^. Indeed, a significant geographical distance-decay pattern of the fungal community was detected in our work. There are two main hypotheses that might explain how environmental factors could affect the microbiota in arthropods. First, theoretically, the microbiota of the host may be acquired from its environmental pool of microbial species, and different host taxa might encounter different microbial pools^38^. Alternatively, some environmental factors (e.g., temperature) could act as a filter providing selection pressure for host microbiomes, which directly or indirectly affect host microbial composition. For instance, our previous study showed that some dominant symbionts, such as *Wolbachia*, were highly sensitive to extreme temperature^21,48,49^; these symbionts reshaped the structure of microbiota communities^20,50^. It remains unclear which of these hypotheses better apply to fungal community differences among stonefly species from different environments. Furthermore, a major portion of community variation (89%) could not be explained by any variables involved in this study (Fig. 4), suggesting that other environmental factors are involved in shaping stonefly fungal community composition. For instance, several previous studies have demonstrated that diet also plays a substantial role in microbial diversity and composition^8,16–19^. Given that the diet of different stonefly species varies significantly from pollen to leaf fragments, detritus, lichen, and animal matter^42–44,51^, it could be speculated that host diet might explain certain aspects of microbiome diversity and composition among stonefly taxa. Further investigation is required to explore these possibilities.

In most cases, many host-related and environmental factors work not independently but in interaction with each other. In particular, aquatic insects are more susceptible to geological changes than their terrestrial counterparts due to their specialized ecological needs and habit range^52^. In the present study, SEM results suggested that a significant negative effect of environmental factors on stoneflies might indirectly impact the stonefly fungal community composition (Fig. 4a). We also found that environmental factors were a relatively strong driver of the stonefly fungal community compare to host condition, similar to patterns recently reported in a broader range of arthropods^9–11^, but in contrast to observations made in some small mammals^13^. For instance, previous studies on the gut microbiota of beetles and fish and on microbiota of the house fly suggested that environmental factors, including food habit, host habitat, or geographical location are more important than host species in shaping microbiota composition^9–11^. In contrast, Knowles et al.^13^ suggest that species identity dominates over the environment in shaping microbiota composition in small mammals. One possible explanation for this might be the highly variable mechanisms of microbial community assembly among different biological systems^19,29–31^. We cannot draw final conclusions from our limited sampling of some stonefly families, and further studies are required to assess the generality of patterns observed here across different host taxa and microbial community types.

### Mechanisms of fungal community assembly in stoneflies

Neutral and null models revealed that deterministic processes dominate over stochastic ones in driving the fungal communities in stoneflies (Figs. 5 and 6). Our findings correspond with the studies of microbial assembly in wild *Drosophila*, in which deterministic forces play a substantial role in community structure^32^. Of the ecological processes assessed, the influences of dispersal limitation, homogenizing dispersal and homogeneous selection were relatively weak, while variable selection and drift were the major deterministic and stochastic processes driving the stonefly fungal community, respectively (Fig. 6). Variable selection may result in the microbiota communities diversifying among distinct environmental conditions, whereas drift disperses communities^7,36,53^. Drift was the main stochastic process driving stonefly fungal communities in our study, a similar result to reports on bacterial communities in the honeybee^33^. The core fungal genus *Aspergillus* found in the wild stoneflies in our work has also been detected in other insects^39,40^, and some of its members are also found in the surrounding environment to the host, such as in the air, soil, and on plants^54^. Thus, we raise a possibility that the fungal microbiota associated with stoneflies may be acquired through stochastic drift from the surrounding environment in which the host resides^38^. This calls for further study that utilizes paired samples of stoneflies and their environment (i.e., water, diet, or soil).

Interestingly, the relative contribution of drift varied drastically among different families. In particular, its relative contribution was much higher for Capniidae and Perlodidae than in Styloperlidae, Leuctridae, and Choloroperlidae. This partly explains why the fungal networks from Capniidae species exhibited more interconnection compared to other stonefly families. In congruence with recent work that revealed the effect of geographic variables on ecological processes^33^, our results also find a significant link between fungal community assembly and environmental factors such as altitude, latitude, longitude, AMT, and AP. Thus, the strength of ecological processes driving fungal communities towards either divergence or convergence is dependent on environmental factors and associated evolutionary history. Fungal community-specific patterns may partly reflect historical population processes as well as ecological effects. From a long-term co-evolution perspective, our findings correspond with previous studies on honeybee bacterial communities, suggesting that stochastic processes are the dominant forces driving co-evolution, and deterministic processes determine the direction of co-evolution^33^.

In summary, our results provide a comprehensive overview of fungal microbiota diversity and composition in stoneflies. We highlight that both host attributes and environmental conditions shape the fungal community in stoneflies by altering the relative influence of community assembly processes. The findings expand our current understanding of the mechanisms underlying microbial community assembly in aquatic insects. Further work is needed to explore the specific function of the identified fungal species and elucidate functional succession of the fungal communities under evolutionary and ecological timescales.

## Methods

### Stoneflies collection and environmental properties

Nymph and adult stoneflies were collected from streams, lakes, and nearby vegetation in multiple locations across China (Fig. 1, Supplementary Table 1). All specimens were preserved in 100% ethanol and stored at −20 °C until DNA extraction. Stonefly species were distinguished based on morphological examination. All samples were deposited in the Institute of Applied Entomology, Yangzhou University, Yangzhou, China. In total, 155 individuals representing 52 populations were obtained, and were identified to 44 species representing 20 genera from eight families within Plecoptera for subsequent microbial analyses. Climatic data (e.g., annual mean temperature (AMT) and annual mean precipitation (AP)) for each sampling location were obtained from the Climate Datasets (https://psl.noaa.gov) (Supplementary Table 1).

### DNA extraction, PCR amplification and sequencing

The stoneflies were surface sterilized by washing individuals with 75% ethanol and then sterile water three times prior to DNA extraction. Total DNA of the individual was extracted using a DNeasy blood and tissue kit (Qiagen, Hilden, Germany) according to the manufacturer’s protocols. The quality and concentration of DNA were evaluated with a 1% agarose gel and a NanoDrop 2000 spectrophotometer (Thermo Scientific, Waltham, MA, USA), and all DNA samples were diluted to the same concentration (10 ng/μL) for subsequent analysis.

The fungal internal transcribed spacer (ITS) ITS1-ITS2 region was amplified with the primers ITS1F (5’-CTTGGTCATTTAGAGGAAGTAA-3’) and ITS2R (5’-GCTGCGTTCTTCATCGATGC-3’)^39^. PCR amplification was carried out in 20 μL reaction mixtures containing 0.4 μL of TransStart FastPfu DNA polymerase (TransGen, Biotech, China), 4 μL of 5× FastPfu buffer, 2 μL of dNTPs (2.5 mM), 0.8 μL of each primer (5 μM), 1 μL of template DNA (10 ng/μL), and 11 μL of ddH_2_O. The program for PCR amplification consisted of DNA pre-denaturation for 5 min at 95 °C, then 30 cycles of 30 s at 95 °C, 30 s at 52 °C, and 45 s at 72 °C, followed by a final extension at 72 °C for 10 min. Negative controls were always performed to ensure there was no contamination. Most samples produced single PCR bands of ~306 bp (Supplementary Fig. 3), and only these were extracted from 2% agarose gels and purified using the AxyPrep DNA Gel Extraction Kit (Axygen Biosciences, Union City, CA, USA) following manufacturer’s instructions. High throughput sequencing of fungal ITS genes was performed on the Illumina PE250 platform (2 × 250 paired ends) (Illumina, CA, USA).

### Bioinformatic analysis

The acquired sequences were filtered for quality control using standardized procedures (Shanghai BIOZERON Co., Ltd., Shanghai, China). The filtering and assembly of raw sequences were carried out using Quantitative Insights Into Microbial Ecology (QIIME version 1.9.0 http://qiime.org/scripts/assign_taxonomy.html)^55^. Raw FASTQ data were demultiplexed and filtered to select for high quality reads from each sample under the following criteria: (i) Reads were removed if any site had an average quality score <20 over a 50 bp sliding window. Reads containing Ns or with a length <50 were also removed. (ii) The remaining pair-end reads of the individual samples were merged into a single fasta file according to their overlaps, with a minimum overlap length of 10 bp. (iii) The maximum mismatch ratio allowed in the overlapping area of the merged sequences was 0.2. (iv) The directionality of reads was corrected based on their barcodes and primer sequences, with no mismatches allowed in the barcode and 2 mismatches allowed in the primers. The sequences were assigned to operational taxonomic units (OTUs) at the 97% similarity threshold using UPARSE version 7.1 (http://drive5.com/uparse/) and chimeric sequences were identified and removed using UCHIME^56^. The phylogenetic affiliation of each ITS gene sequence was identified with the UCLUST algorithm (http://www.drive5.com/usearch/manual/uclust_algo.html) against the UNITE database (UNITE version 8.2) with a confidence threshold of 80%. A total of 77.47% OTUs were identified as fungal, and 22.53% OTUs were non-fungal.

### Statistical analysis

#### Fungal diversity analysis

All analyses were carried out in R version 4.0.5^57^. A normalized number of sequences were randomly extracted from each sample to calculate alpha diversity indices that were estimated with the ‘vegan’ package. Nonparametric statistical tests were used to detect significant differences in the Shannon diversity index and richness among different stonefly families or ontogeny stages with the ‘EasyStat’ package. Before the calculation of beta diversity, relative abundances were used to standardize the OTU profiles. Bray-Curtis similarity matrices were prepared using the ‘vegan’ package. A perMANOVA^58^ (Adonis, transformed data by Bray-Curtis, permutation = 999) was used to test if the beta diversity differed among treatments. Then, principal coordinate analysis (PCoA) plots and t-Distributed stochastic neighbor embedding (t-SNE) were generated according to Bray-Curtis similarity matrices created using the package “ggplot2”. t-Distributed stochastic neighbor embedding (t-SNE) analysis was performed with the package ‘t-sne’.

#### Distance-decay relationships

Pairwise geographic distances between samples were calculated from the latitude and longitude coordinates using the ‘geosphere’ package in R. The microbial raw data was normalized by TMM (trimmed mean of means) with the ‘edgeR’ package, and then Mantel tests were conducted to calculate the Spearman distances using the ‘vegan’ package. Then, the distance-decay rates of the fungal communities were calculated as the slopes of ordinary least-squares regressions between geographic distance and community similarity.

#### Network analysis

Fungal co-occurrence networks were constructed to reveal significant relationships between the relative abundance of OTUs in the fungal community of each stonefly family. Co-occurrence networks were constructed using the ‘SpiecEasi’ package and plotted using ‘ggClusterNet’^59^. Robust correlations with Spearman’s correlation coefficients >0.6 and false discovery rate-corrected *p*-values < 0.05 were considered to be statistically significant. To describe the topology of the networks, a set of metrics, including average degree, average path length, clustering coefficient, network diameter, and centralization degree were calculated using the ‘vegan’ and ‘igraph’ packages^60^. Network complexity is reflected in the parameter ‘average degree’, where a higher average degree represents a greater network complexity^61^. To assess nonrandom patterns in the resulting networks, we compared our network against its randomized version generated using the ‘igraph’ package.

#### Evaluating the impact of the host-related and environmental variables on fungal community

To examine the effect of host-related and environmental variables on stonefly fungal communities, we first calculated the relative contribution of host-related factors, environmental factors, and their combined effects on stonefly fungal community compositions with a variance partitioning analysis (VPA) using the ‘vegan’ package. The significance of partitioned fractions was tested by performing a permutation test for distance-based redundancy analysis using the function *anova.cca* from the ‘vegan’ package^62^. Then, a structural equation model (SEM)^63^ was constructed to explore the causal relationships among host attributes, environmental variables, and fungal community composition. The SEM tests were performed using the ‘SEM’ package in R. As non-normal distribution of variables may compromise SEM analyses results, we also conducted Mantel tests using the Spearman method with 1000 permutations to determine the associations between microbial community structure variation and both host-related and environmental factors.

Finally, to estimate the degree of autocorrelation in a set of environmental factors, Mantel tests using Pearson’s r for each environmental factor were performed with the ‘vegan’ package. Canonical correspondence analysis (CCA) was also conducted using ‘vegan’ to determine relative contributions of each host-related or environmental variable to the overall compositional variation in the microbiota communities, and results were visualized with the ‘ggplot2’ package^64^.

#### Fungal community assembly analyses

Two approaches were used to infer the stonefly fungal community assembly process. Firstly, the Sloan neutral model^65^ was applied to assess the importance of the neutral processes in the assembly of stonefly communities for all samples and separately for each stonefly family, using the R code from Burns et al.^66^. Specifically, the neutral model uses the average abundance of each OTU across all stonefly individuals (mean relative abundance) to predict the occurrence frequency of each OTU in the metacommunity. The neutral model was generated using the *pbeta* function in the ‘stats’ package and fit to data using the *nlsLM* function from ‘minpack.lm’. The 95% confidence interval was determined using the *binconf* function in the ‘Hmisc’ package^67^. In the model, the goodness of fit to the neutral model was assessed using *R*^*2*^ as the coefficient of determination. The estimated migration rate (*m*) represents the probability of random loss of an OTU in a local community replaced by dispersal from the metacommunity, and can thus be interpreted as a measure of dispersal limitation.

Secondly, the null model was used to evaluate phylogenetic patterns in stonefly fungal communities through calculating the beta Nearest Taxon Index (βNTI) between pairs of samples^53^. A fungal phylogenetic tree was constructed using ghost-tree^68–70^ (https://github.com/JTFouquier/q2-ghost-tree). Meanwhile, βNTI was estimated by comparing the observed β-mean nearest taxon distance (βMNTD) with the mean of a null distribution of βMNTD (999 randomizations), and by normalizing its standard deviation using the ‘picante’ package in R. We subsequently incorporated βNTI and the Raup-Crick index (RCI) to estimate the relative strength of different ecological processes in driving the composition of fungal communities: the relative impact of community turnover regulated by deterministic processes— heterogeneous selection and homogeneous selection—can be indicated by the proportion of sample pairs with βNTI values > 2 and βNTI < −2, respectively. Conversely, stochasticity was recognized to impact community pairs that fell within |βNTI | < 2. To discern stochastic processes, including homogenizing dispersal (mass effect), dispersal limitation, and drift, a Bray-Curtis based RCI was calculated with RCI > 0.95, |RCI | < 0.95 and RCI < − 0.95 being interpreted as dispersal limitation, drift, and homogenizing dispersal, respectively. To assess the major factors that affected assembly processes, a Mantel test based on Spearman’s correlation coefficients was conducted to compare the βNTI values with the Euclidean distance matrices for each variable using the ‘vegan’ package in R.

### Reporting summary

Further information on research design is available in the Nature Research Reporting Summary linked to this article.

## Supplementary information

Reporting Summary

Supplementary Material

## Data Availability

All sequencing data reported in this paper are available at the Sequence Read Archive (SRA) under BioProject number PRJNA772541.

## Code availability

R code used for data processing, graph creation, and statistical analyses related to this paper are available on GitHub (https://github.com/taowenmicro/2021_isecet_geographical_distribution).
