# Supplementary material for: Mechanisms of fungal community assembly in wild stoneflies moderated by host characteristics and local environment
Source: NPJ Biofilms Microbiomes. 2022 Apr 27;8:31. doi: 10.1038/s41522-022-00298-9 (PMC9046381; doi:10.1038/s41522-022-00298-9)
Supplement: Supplementary file 1 — Reporting Summary [file 41522_2022_298_MOESM1_ESM.pdf]

nature portfolio

Corresponding author(s): DBPR_NPJBIOFILMS-01517

Last updated by author(s): Jan 15, 2022

Reporting Summary

Nature Portfolio wishes to improve the reproducibility of the work that we publish. This form provides structure for consistency and transparency in reporting. For further information on Nature Portfolio policies, see our Editorial Policies and the Editorial Policy Checklist.

Statistics

For all statistical analyses, confirm that the following items are present in the figure legend, table legend, main text, or Methods section.

n/a	Confirmed	
☐	☒	The exact sample size (*n*) for each experimental group/condition, given as a discrete number and unit of measurement
☐	☒	A statement on whether measurements were taken from distinct samples or whether the same sample was measured repeatedly
☐	☒	The statistical test(s) used AND whether they are one- or two-sided<br>*Only common tests should be described solely by name; describe more complex techniques in the Methods section.*
☐	☒	A description of all covariates tested
☐	☒	A description of any assumptions or corrections, such as tests of normality and adjustment for multiple comparisons
☐	☒	A full description of the statistical parameters including central tendency (e.g. means) or other basic estimates (e.g. regression coefficient) AND variation (e.g. standard deviation) or associated estimates of uncertainty (e.g. confidence intervals)
☐	☒	For null hypothesis testing, the test statistic (e.g. *F*, *t*, *r*) with confidence intervals, effect sizes, degrees of freedom and *P* value noted<br>*Give P values as exact values whenever suitable.*
☒	☐	For Bayesian analysis, information on the choice of priors and Markov chain Monte Carlo settings
☒	☐	For hierarchical and complex designs, identification of the appropriate level for tests and full reporting of outcomes
☐	☒	Estimates of effect sizes (e.g. Cohen's *d*, Pearson's *r*), indicating how they were calculated

Our web collection on statistics for biologists contains articles on many of the points above.

Software and code

Policy information about availability of computer code

Data collection	High throughput sequencing of fungal ITS genes was performed on the Illumina PE250 platform (2 × 250 paired ends) (Illumina, CA, USA).The filtering and assembly of raw sequences were carried out using Quantitative Insights Into Microbial Ecology (QIIME version 1.9.0 http://qiime.org/scripts/assign_taxonomy.html).
Data analysis	All analyses were carried out in R version 4.0.5.

For manuscripts utilizing custom algorithms or software that are central to the research but not yet described in published literature, software must be made available to editors and reviewers. We strongly encourage code deposition in a community repository (e.g. GitHub). See the Nature Portfolio guidelines for submitting code & software for further information.

Data

Policy information about availability of data

All manuscripts must include a data availability statement. This statement should provide the following information, where applicable:

- Accession codes, unique identifiers, or web links for publicly available datasets
- A description of any restrictions on data availability
- For clinical datasets or third party data, please ensure that the statement adheres to our policy

All sequencing data reported in this paper are available at the Sequence Read Archive (SRA) under BioProject number PRJNA772541 (https://www.ncbi.nlm.nih.gov/bioproject/PRJNA772541/).

Field-specific reporting

Please select the one below that is the best fit for your research. If you are not sure, read the appropriate sections before making your selection.

☒ Life sciences ☐ Behavioural & social sciences ☐ Ecological, evolutionary & environmental sciences

For a reference copy of the document with all sections, see nature.com/documents/nr-reporting-summary-flat.pdf

Life sciences study design

All studies must disclose on these points even when the disclosure is negative.

Sample size	In total, the study included 155 samples.
Data exclusions	No data were excluded from the analyses.
Replication	All sequencing data and R code used for data analyses are available at the Sequence Read Archive (SRA) (BioProject number PRJNA772541) and GitHub, respectively. All attempts at replication were successful.
Randomization	Stonefly species were distinguished based on morphological examination. In total, 155 individuals representing 52 populations were obtained, and were identified to 44 species representing 20 genera from eight families within Plecoptera for microbial analyses.
Blinding	All authors approved group allocation during data collection and analysis.

Reporting for specific materials, systems and methods

We require information from authors about some types of materials, experimental systems and methods used in many studies. Here, indicate whether each material, system or method listed is relevant to your study. If you are not sure if a list item applies to your research, read the appropriate section before selecting a response.

Materials & experimental systems

n/a	Involved in the study	
☒	☐	Antibodies
☒	☐	Eukaryotic cell lines
☒	☐	Palaeontology and archaeology
☐	☒	Animals and other organisms
☒	☐	Human research participants
☒	☐	Clinical data
☒	☐	Dual use research of concern

Methods

n/a	Involved in the study	
☒	☐	ChIP-seq
☒	☐	Flow cytometry
☒	☐	MRI-based neuroimaging

Animals and other organisms

Policy information about studies involving animals; ARRIVE guidelines recommended for reporting animal research

Laboratory animals	The study did not involve laboratory animals.
Wild animals	Nymph and adult stoneflies were collected from streams, lakes, and nearby vegetation in multiple locations across China. All specimens were preserved in 100% ethanol and stored at -20°C until DNA extraction.
Field-collected samples	The study did not involve samples collected from the field.
Ethics oversight	No ethical approval or guidance was required. The study did not involve any legally protected insect species.

Note that full information on the approval of the study protocol must also be provided in the manuscript.
