# Supplementary material for: Mechanisms of fungal community assembly in wild stoneflies moderated by host characteristics and local environment
Source: NPJ Biofilms Microbiomes. 2022 Apr 27;8:31. doi: 10.1038/s41522-022-00298-9 (PMC9046381; doi:10.1038/s41522-022-00298-9)
Supplement: Supplementary file 2 — Supplementary Material [file 41522_2022_298_MOESM2_ESM.pdf]

Supplementary Information for

Mechanisms of fungal community assembly in wild stoneflies moderated by host characteristics and local environment

Yu-Xi Zhu[1,2], Qing-Bo Huo[1,2], Tao Wen[3], Xin-Yu Wang[1,2], Meng-Yuan Zhao[1,2], Yu-Zhou Du[1,2]*

[1] School of Horticulture and Plant Protection & Institute of Applied Entomology, Yangzhou University, Yangzhou 225009, China

[2] Joint International Research Laboratory of Agriculture and Agri-Product Safety, the Ministry of Education, Yangzhou University, Yangzhou 225009, China

[3] The Key Laboratory of Plant Immunity, Jiangsu Provincial Key Lab for Organic Solid Waste Utilization, Jiangsu Collaborative Innovation Center for Solid Organic Wastes, Educational Ministry Engineering Center of Resource-saving fertilizers, Nanjing Agricultural University, Nanjing 210095, China

***Author for correspondence**: Yu-Zhou Du

E-mail: yzdu@yzu.edu.cn

Running Head: Fungal community assembly in wild stoneflies

Supplementary Figures

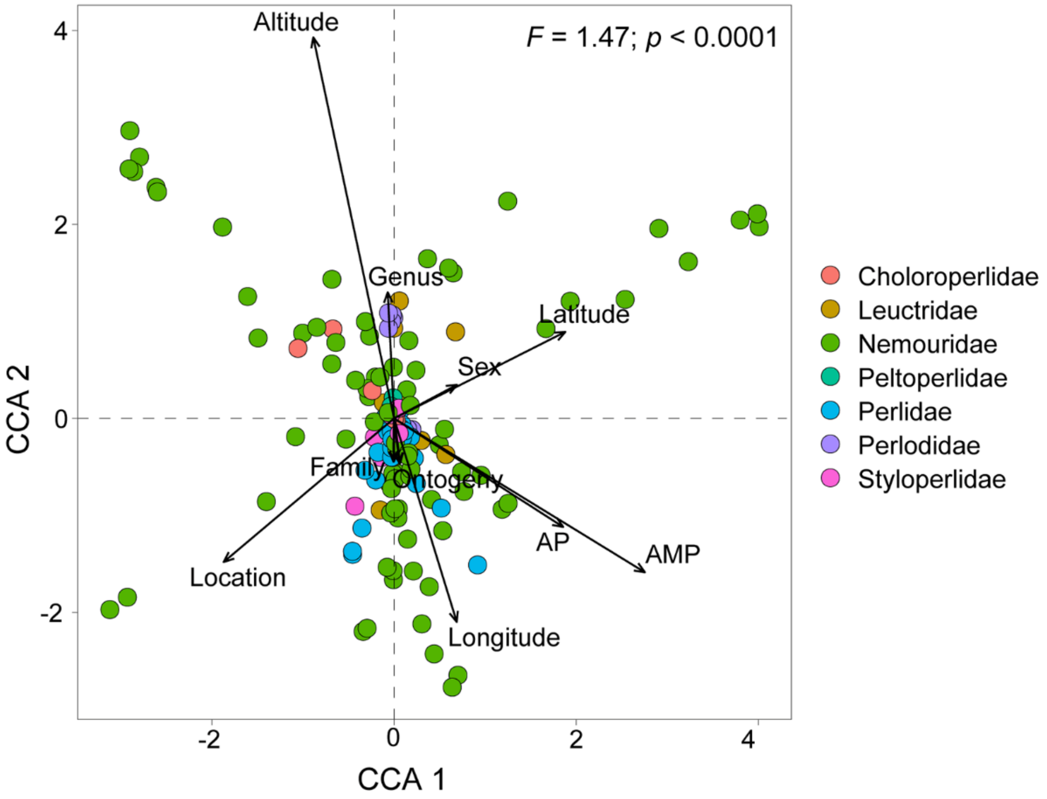

Supplementary Fig. 1 Canonical correspondence analysis (CCA) showing the relative contribution of each host-related or environmental variable to the overall compositional variation in the mycological communities. Differently coloured data points represent different stonefly families.

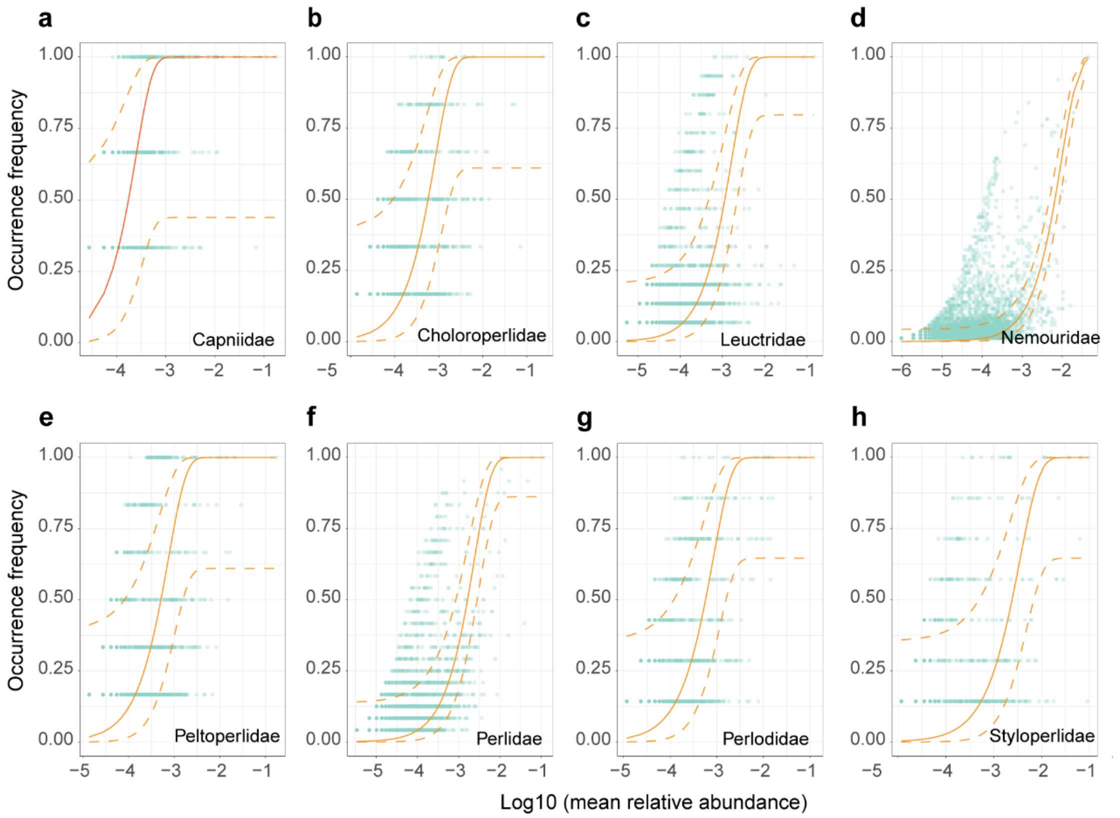

Supplementary Fig. 2 Fit of the neutral model for each stonefly family: a Capniidae, **b** Choloroperlidae, **c** Leuctridae, **d** Nemouridae, **e** Peltoperlidae, **f** Perlidae, **g** Perlodidae, and **h** Styloperlidae. The yellow solid and dashed lines indicate the predicted occurrence and 95% confidence intervals of the neutral model, respectively.

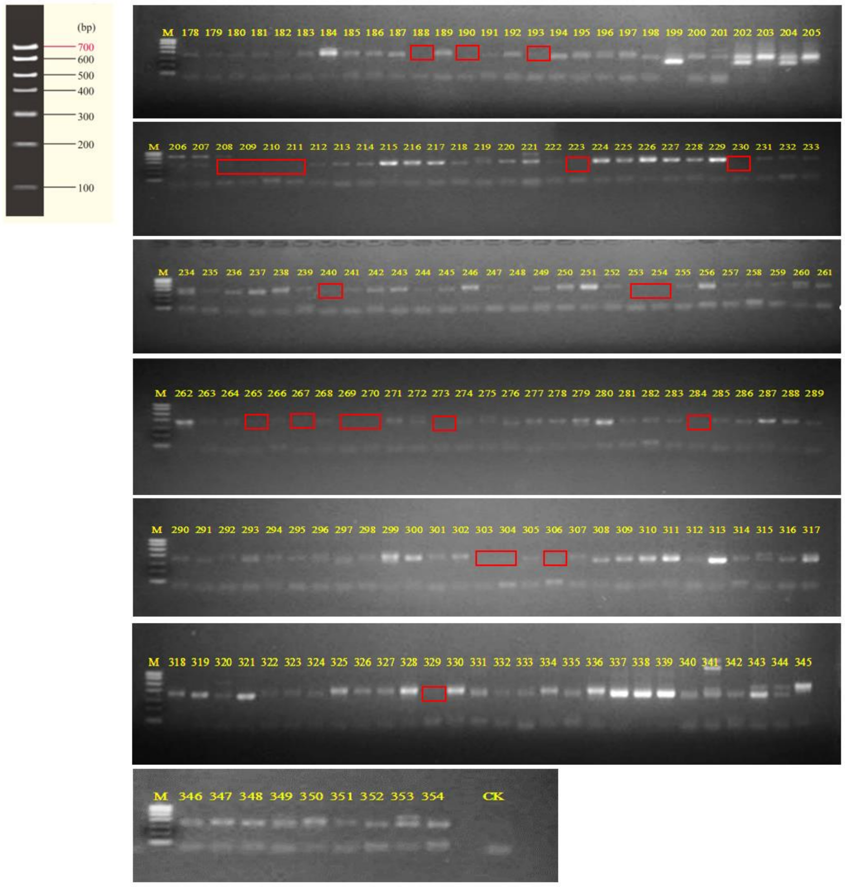

Supplementary Fig. 3 Raw gel images of ITS amplicon products from each sample. Left panel shows the marker used in the gel. Samples marked in red boxes in the figure were excluded from the study.

Supplementary Tables

Supplementary Table 1. Sample information of wild stoneflies examined in this study

No.	Population	Species	Genus	Family	Individual (Male (♂), Female (♀); Adult (A), Nymph (N))	Location	Longitude	Latitude	Altitude (m)	AMT[1] (℃)	AP[2] (mm)
1	CDr	*Cryptoperla dui* Sivec, 2005	*Cryptoperla*	Peltoperlidae	3 (2♂, 1♀; 3 A)	Chongzhou, Sichuan	30.80	103.21	1598	10.56	16.62
2	MQi	*Microperla qinlinga* Chen	*Microperla*	Peltoperlidae	3 (1♂, 2♀; 3 A)	Xian, Shanxi	33.84	107.83	1280	8.77	20.72
3	StSt	*Styloperla starki* Zhao, Huo & Du	*Styloperla*	Styloperlidae	3 (3♂, 0♀; 3 A)	Hangzhou, Zhejiang	30.33	119.44	571	14.78	29.14
4	StSp_JX	*Styloperla spinicercia* Wu, 1935	*Styloperla*	Styloperlidae	1 (1♂, 0♀; 1 A)	Yichun, Jiangxi	28.54	114.61	280	16.97	33.18
5	StSp_FJ	*Styloperla spinicercia* Wu, 1935	*Styloperla*	Styloperlidae	3 (2♂, 1♀; 3 A)	Shanming, Fujian	26.80	117.05	274	16.13	30.97
6	IAa	*Isoperla asiatica* Raušer	*Isoperla*	Perlodidae	5 (1♂, 4♀; 5 A)	Hinggan League, Inner Mongolia	47.28	120.48	684	1.06	25.23
7	SMa	*Stavsolus manchuricus* Teslenko	*Stavsolus*	Perlodidae	2 (0♂, 2♀; 2 A)	Benxi, Liaoning	41.18	124.67	589	6.80	15.34
8	ONi	*Oyamia nigribasis* Banks	*Oyamia*	Perlidae	7 (1♂, 3♀, 3 unknown; 4 A, 3 N)	Benxi, Liaoning	41.18	124.67	589	6.80	15.34
9	TSp.	*Togoperla sp.*	*Togoperla*	Perlidae	3 (2♂, 1♀; 3 A)	Nanping, Fujian	27.75	117.69	230	15.87	30.97
10	TPe	*Togoperla perpicta* Klap ǎlek	*Togoperla*	Perlidae	2 (2♂, 0♀; 2 A)	Huangshan, Anhui	30.09	118.14	597	14.78	28.40
11	NSp.	*Neoperla sp.*	*Neoperla*	Perlidae	3 (2♂,1♀; 3 A)	Huangshan, Anhui	30.09	118.14	597	14.78	28.40
12	NJi	*Neoperla jiangsuensis* Chen & Du	*Neoperla*	Perlidae	6 (3♂, 0♀, 3 unknown; 3 A, 3 N)	Huaian, Jiangsu	32.44	118.28	145	14.20	25.23
13	FSp.	*Flavoperla sp.*	*Flavoperla*	Perlidae	3 (0♂,3♀; 3 A)	Shaoguan, Guangdong	24.69	114.17	635	19.93	34.45
14	SuSp.1	*Suwallia sp.1*	*Suwallia*	Choloroperlidae	3 (0♂,3♀; 3 A)	Benxi, Liaoning	41.18	124.67	705	6.80	15.34
15	SuSp.2	*Suwallia sp.2*	*Suwallia*	Choloroperlidae	3 (1♂,2♀; 3 A)	Hulun Buir, Inner Mongolia	51.46	122.56	860	-2.29	9.63
16	PMa_NX	*Protonemura macrodactyla* Du et Zhou, 2007	*Protonemura*	Nemouridae	4 (2♂,2♀; 4 A)	Guyuan, Ningxia	35.34	106.35	2130	8.77	15.56

17	PMa_HB	*Protonemura macrodactyla* Du et Zhou, 2007	*Protonemura*	Nemouridae	3 (2♂,1♀; 3 A)	Shennongjia Forestry District, Hubei	31.48	110.32	2500	11.86	19.55
18	PBi	*Protonemura biintrans* Li et Yang,2008	*Protonemura*	Nemouridae	3 (2♂,1♀; 3 A)	Baoji, Shanxi	34.04	107.01	1336	20.33	31.31
19	SpSo	*Sphaeronemoura songshana* Li et Yang	*Sphaeronemoura*	Nemouridae	1 (1♂,0♀; 1 A)	Zhangjiakou, Hebei	39.98	115.40	1300	10.13	12.02
20	SpSp.	*Sphaeronemoura sp.*	*Sphaeronemoura*	Nemouridae	3 (3 unknown; 3 N)	Leishan, Guizhou	26.38	108.08	1450	16.43	27.67
21	MMu	*Mesonemoura multispira* (Wu, 1973)	*Mesonemoura*	Nemouridae	3 (3♂,0♀; 3 A)	Kangding, Sichuan	29.99	101.89	2920	2.81	16.62
22	MSi	*Mesonemoura sichuanensis* Du & Ji, 2015	*Mesonemoura*	Nemouridae	3 (1♂,2♀; 3 A)	Luding, Sichuan	29.88	102.02	3646	2.89	16.62
23	MSp	*Mesonemoura spiroflagellata* (Wu, 1973)	*Mesonemoura*	Nemouridae	2 (1♂,1♀; 2 A)	Ziyang, Sichuan	29.85	105.51	3100	15.55	29.79
24	IBa	*Indonemoura baishanzuensis* Li et Yang	*Indonemoura*	Nemouridae	3 (2♂,1♀; 3 A)	Huzhou, Zhejiang	30.61	119.87	411	14.78	29.14
25	ISp.	*Indonemoura sp.*	*Indonemoura*	Nemouridae	2 (2♂,0♀; 2 A)	Huzhou, Zhejiang	30.61	119.87	411	14.78	29.14
26	IGu	*Indonemoura guangdongensis* Li et Yang, 2006	*Indonemoura*	Nemouridae	2 (0♂,2♀; 2 A)	Shaoyang, Hunan	26.69	110.62	1380	17.45	36.26
27	ISc	*Indonemoura scalprata* (Li & Yang, 2007)	*Indonemoura*	Nemouridae	2 (1♂,1♀; 2 A)	Qingyuan, Guangdong	24.60	113.56	133	19.93	35.21
28	ICu	*Indonemoura curvispina* Li &Yang, 2017	*Indonemoura*	Nemouridae	2 (0♂,2♀; 2 A)	Baoshan, Yunnan	25.37	98.21	2196	31.31	28.44
29	AAn	*Amphinemura annulata* Du & Ji, 2014	*Amphinemura*	Nemouridae	2 (1♂,1♀; 2 A)	Guyuan, Ningxia	35.34	106.35	2040	8.77	15.56
30	Ach_AH	*Amphinemura chui* (Wu)	*Amphinemura*	Nemouridae	3 (2♂,1♀; 3 A)	Chizhou, Anhui	30.48	117.82	880	14.78	28.40
31	Ach_ZJ	*Amphinemura chui* (Wu)	*Amphinemura*	Nemouridae	4 (2♂,2♀; 4 A)	Huzhou, Zhejiang	30.61	119.87	278	14.78	29.14
32	ALl	*Amphinemura lii* Zhu et Yang	*Amphinemura*	Nemouridae	3 (0♂,3♀; 3 A)	Linzhi, Xizang	29.23	95.17	780	1.87	8.59
33	ANi_QH	*Amphinemura ningxiana* Li et Yang, 2011	*Amphinemura*	Nemouridae	3 (3♂,0♀; 3 A)	Yushu, Qinghai	32.54	96.66	4311	17.07	12.01
34	ANi_SC	*Amphinemura ningxiana* Li et Yang, 2011	*Amphinemura*	Nemouridae	3 (3♂,0♀; 3 A)	Dazhou, Sichuan	30.86	106.97	286	13.67	27.57
35	ATu	*Amphinemura tubulata* Du et Zhou	*Amphinemura*	Nemouridae	1 (0♂,1♀; 1 A)	Kangding, Sichuan	30.06	101.58	3523	2.89	7.22
36	ADi_SX	*Amphinemura didyma* Zhu et Yang	*Amphinemura*	Nemouridae	2 (2♂,0♀; 2 A)	Baoji, Shanxi	34.03	107.87	1900	20.33	31.31
37	ADi_HN	*Amphinemura didyma* Zhu et Yang	*Amphinemura*	Nemouridae	3 (2♂,1♀; 3 A)	Luoyang, Henan	33.74	111.73	1100	12.69	23.70

38	ATi	*Amphinemura tianmushana* Li et Yang	*Amphinemura*	Nemouridae	4 (4♂,0♀; 4 A)	Huzhou, Zhejiang	30.42	119.44	263	14.78	29.14
39	ASi	*Amphinemura sinensis* (Wu, 1926)	*Amphinemura*	Nemouridae	7 (1♂,3♀; 3 unknown; 4 A, 3 N)	Liangyungang, Jiangsu	34.64	119.33	113	13.60	20.58
40	NMe	*Nemoura meniscata* Li et Yang	*Nemoura*	Nemouridae	3 (2♂,1♀; 3 A)	Baoji, Shanxi	34.03	107.87	1396	20.33	31.31
41	NPa	*Nemoura papilla* Okamoto, 1922	*Nemoura*	Nemouridae	2 (2♂,0♀; 2 A)	Luoyang, Henan	34.72	111.87	1500	11.49	18.83
42	NTr	*Nemoura tridenticula* Li, Wang et Yang	*Nemoura*	Nemouridae	3 (2♂,1♀; 3 A)	Deqin, Yunnan	28.29	99.16	3267	5.88	11.59
43	NNa	*Nemoura nankinensis* Wu, 1926	*Nemoura*	Nemouridae	3 (1♂,2♀; 3 A)	Nanjing, Jiangsu	32.06	118.86	46	14.54	25.23
44	NGu_JS	*Nemoura guangdongensis* Li et Yang	*Nemoura*	Nemouridae	3 (0♂,3♀; 3 A)	Yixing, Jiangsu	31.22	119.80	58	14.54	29.14
45	NGu_ZJ	*Nemoura guangdongensis* Li et Yang	*Nemoura*	Nemouridae	2 (0♂,2♀; 2 A)	Huzhou, Zhejiang	30.42	119.44	1308	14.78	29.14
46	NSi	*Nemoura sichuanensis* Li et Yang, 2006	*Nemoura*	Nemouridae	3 (2♂,1♀; 3 A)	Luding, Sichuan	29.88	102.02	3646	2.89	16.62
47	PSm	*Perlomyia smithae* Nelson et Hanson,1973	*Perlomyia*	Leuctridae	3 (0♂,3♀; 3 A)	Benxi, Liaoning	41.18	124.67	589	6.80	15.34
48	RSi_HNCZ	*Rhopalopsole sinensis* Yang & Yang, 1993	*Rhopalopsole*	Leuctridae	3 (1♂,2♀; 3 A)	Chenzhou, Hunan	24.98	112.91	696	17.44	35.21
49	RSi_GX	*Rhopalopsole sinensis* Yang & Yang, 1993	*Rhopalopsole*	Leuctridae	3 (0♂,3♀; 3 A)	Guilin, Guangxi	25.41	111.30	396	17.45	36.26
50	RSi_HNYZ	*Rhopalopsole sinensis* Yang & Yang, 1993	*Rhopalopsole*	Leuctridae	3 (1♂,2♀; 3 A)	Yongzhou, Hunan	25.45	111.33	532	17.45	36.26
51	LFu	*Leuctra fusca* (Linnaeus)	*Leuctra*	Leuctridae	3 (2♂,1♀; 3 A)	Haidong, Qinghai	36.82	102.54	2222	3.77	8.85
52	CBi	*Capnia bilobata* Chen & Song	*Capnia*	Capniidae	3 (1♂,2♀; 3 A)	Baoji, Shanxi	34.03	107.87	1526	20.33	31.31
**Total**	**52**	**44**	**20**	**8**	**155**						

[1]AMT: annual mean temperature; [2]AP: annual mean precipitation

Supplementary Table 2. Explained variation and statistical significance of each host-related or environmental variable on the fungal community assembly in stoneflies

Factors	Explained variation (%)	*F*	*p*
Location	0.88	1.35	0.001
Altitude	1.28	1.76	0.001
Latitude	1.14	1.57	0.001
Longitude	1.10	1.60	0.001
AMT[1]	1.30	1.70	0.001
AP[2]	1.47	1.61	0.001
Family	1.16	1.27	0.007
Genus	1.46	1.31	0.001
Ontogeny	0.92	1.23	0.12
Sex	0.65	1.17	0.027

[1]AMT: annual mean temperature; [2]AP: annual mean precipitation
